# Machine learning analysis for the association between breast feeding and metabolic syndrome in women

**DOI:** 10.1038/s41598-024-53137-6

**Published:** 2024-02-20

**Authors:** Jue Seong Lee, Eun-Saem Choi, Hwasun Lee, Serhim Son, Kwang-Sig Lee, Ki Hoon Ahn

**Affiliations:** 1 Department of Pediatrics, Korea University College of Medicine, Korea University Anam Hospital, Seoul, South Korea; 2 Department of Obstetrics and Gynecology, Korea University College of Medicine, Korea University Anam Hospital, 73 Goryeodae-ro, Seongbuk-gu, Seoul, 02841 South Korea; 3 Department of Biostatistics, Korea University College of Medicine, Seoul, South Korea; 4 AI Center, Korea University College of Medicine, Korea University Anam Hospital, 73 Goryeodae-ro, Seongbuk-gu, Seoul, 02841 South Korea

**Keywords:** Cardiovascular diseases, Metabolic disorders

## Abstract

This cross-sectional study aimed to develop and validate population-based machine learning models for examining the association between breastfeeding and metabolic syndrome in women. The artificial neural network, the decision tree, logistic regression, the Naïve Bayes, the random forest and the support vector machine were developed and validated to predict metabolic syndrome in women. Data came from 30,204 women, who aged 20 years or more and participated in the Korean National Health and Nutrition Examination Surveys 2010–2019. The dependent variable was metabolic syndrome. The 86 independent variables included demographic/socioeconomic determinants, cardiovascular disease, breastfeeding duration and other medical/obstetric information. The random forest had the best performance in terms of the area under the receiver-operating-characteristic curve, e.g., 90.7%. According to random forest variable importance, the top predictors of metabolic syndrome included body mass index (0.1032), medication for hypertension (0.0552), hypertension (0.0499), cardiovascular disease (0.0453), age (0.0437) and breastfeeding duration (0.0191). Breastfeeding duration is a major predictor of metabolic syndrome for women together with body mass index, diagnosis and medication for hypertension, cardiovascular disease and age.

## Introduction

The occurrences of metabolic syndrome and its associated risk factors, like hypertension, dyslipidemia, insulin resistance, and central obesity, have increased over the past few decades^1,2^. The clinical importance of metabolic syndrome has been acknowledged for long time owing to its increased risk for type 2 diabetes and cardiovascular disease (CVD)^3^. There have been considerable research to find the factors reducing the risk of metabolic syndrome. As a series of events following pregnancy, such as delivery and breastfeeding are known to have long-term impacts on women’s health, multiple studies evaluated the association between the pregnancy-related factors and metabolic syndrome^4–6^. Especially, the protective role of breastfeeding received attentions in terms of resetting metabolic change caused by pregnancy which includes insulin resistance and accumulation of lipid^6^. Several studies reported that the breastfeeding was associated with reducing the risk of metabolic syndromes^7,8^. While some studies found no association between breastfeeding and metabolic syndrome^8–10^. In addition, various mediating factors should be considered to determine the association between breastfeeding and metabolic syndrome.

CVD and metabolic syndrome are closely related owing to shared predisposing risk factors^11,12^. The proportion of pregnant women with CVD has increased over the decades^13–15^. Additionally, the number of pregnant women with pregestational comorbidities, like diabetes and obesity, is also on the rise^13,15–17^. These changes are presumably associated with maternal metabolic syndrome, but the validated data is limited^18–20^.

Understanding the association between metabolic syndrome and breastfeeding is important in terms of suggesting another possible prevention of metabolic syndrome. Therefore, we aimed to investigate the association between obstetric characteristics like breastfeeding and metabolic syndrome and the presence of CVD in a large-scale Asian population-based cross-sectional study of women, using artificial intelligence. We developed a prediction model for metabolic syndrome using artificial intelligence, which assessed 86 variables, including general obstetric characteristics (e.g., parity, gravidity), medical information, demographics, dietary preferences, lifestyles, and socioeconomic factors.

## Results

### General obstetric characteristics and metabolic syndrome

**Figure 1:**
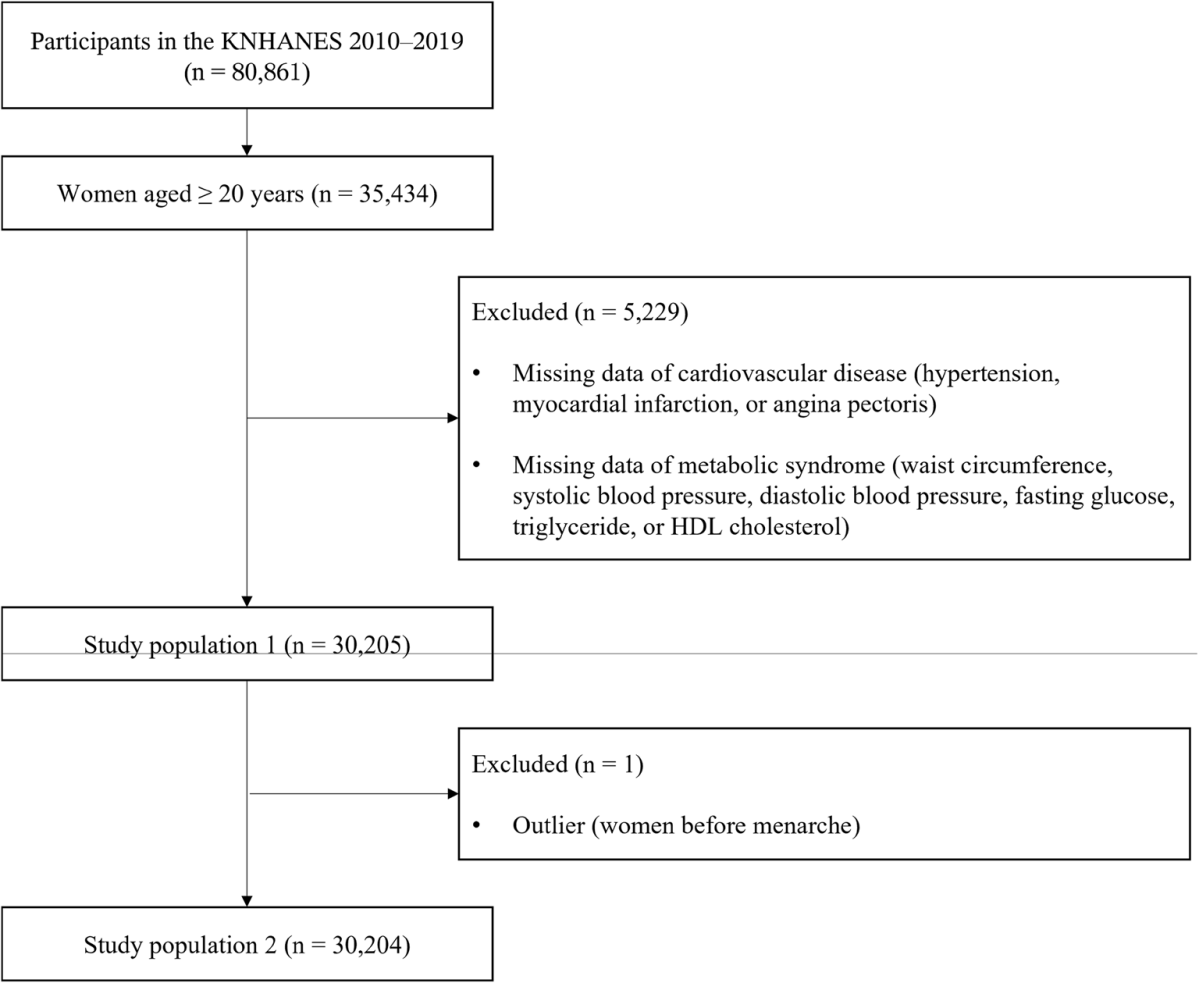
A flow chart summarizing the experimental approach of the study. KNHANES, Korean National Health and Nutrition Examination Survey; HDL, high-density lipoprotein.

Among the 80,861 participants in the KNHANES 2010–2019, only women older than 20 years of age were included (n = 35,434). Patients with missing CVD or metabolic syndrome data were excluded (n = 5229). After excluding the outliers (n = 1), the data of 30,204 participants were analyzed (Fig. 1). The mean age of the participants was 50.93 years, and the prevalence of metabolic syndrome was 28.38% (8571/30,204) (Table 1). Among the study population, 21,865 (72.85%) had a history of breastfeeding. The prevalence of CVD was 23.50% (7097/30,204).

**Table 1:** The baseline characteristics evaluated for the prediction of metabolic syndrome.

Variables	Study population (n = 30,204)
Age at enrollment (years)	50.93 ± 16.14
Body mass index (kg/m^2^)	23.48 ± 3.56
Waist circumference (cm)	79.09 ± 9.90
Systolic blood pressure (mmHg)	117.30 ± 17.84
Diastolic blood pressure (mmHg)	73.78 ± 9.72
Fasting glucose (mg/dL)	97.81 ± 21.70
Triglycerides (mg/dL)	115.90 ± 79.55
HDL cholesterol (mg/dL)	53.67 ± 12.53
Age at menarche (years)	14.37 ± 2.14
Menstrual status, n (%)	
Menstruation	13,976 (46.51%)
Pregnant	211 (0.70%)
Breast-feeding	318 (1.06%)
Menopause	15,203 (50.59%)
Others	344 (1.14%)
Married, n (%)	26,448 (87.58%)
Nulligravida, n (%)	4290 (14.20%)
Gravidity	3.34 ± 2.38 (3.00)
Parous women, n (%)	25,320 (84.29%)
Breastfeeding experience, n (%)	21,865 (72.85%)
Number of breastfed children	1.80 ± 1.57 (2.00)
Breastfeeding duration (months)	25.11 ± 34.49 (13.00)
Use of oral contraceptive, n (%)	5035 (16.75%)
Cardiovascular disease, n (%)	7097 (23.50%)
Hypertension, n (%)	6855 (22.70%)
Myocardial infarction, n (%)	175 (0.58%)
Angina, n (%)	563 (1.86%)
Major depressive disorder, n (%)	3282 (10.87%)
Stroke, n (%)	547 (1.81%)
Kidney failure, n (%)	102 (0.34%)
Antihypertensive drug	6429 (21.29%)
Drug treatment for glucose control	2199 (7.28%)
Insulin	203 (0.67%)
Oral hypoglycemic agents	2143 (7.10%)
Lipid-lowering agent	3443 (11.40%)
LDL cholesterol (mg/dL)	115.51 ± 32.97
Total cholesterol (mg/dL)	192.36 ± 36.84
White blood cell counts (Thous/µL)	5.87 ± 1.66
Red blood cell counts (Mil/µL)	4.34 ± 0.35
Hematocrit (%)	39.78 ± 3.12
Hemoglobin (g/dL)	13.10 ± 1.15
Serum creatinine (mg/dL)	0.72 ± 0.20
Blood urea nitrogen (mg/dL)	14.05 ± 4.51
Daily intake of calories (kcal)	1687.99 ± 687.09
Daily intake of fat (g)	35.51 ± 27.51
Daily intake of water (g)	929.09 ± 566.30
Daily intake of vitamin C (mg)	89.43 ± 105.50
Daily intake of sodium (mg)	3373.46 ± 2378.88
Daily intake of calcium (mg)	459.05 ± 304.05
Daily intake of carbohydrate (g)	277.05 ± 113.80
Daily intake of iron (mg)	12.97 ± 10.53
Daily intake of potassium (mg)	2716.56 ± 1498.10
Daily intake of protein (g)	59.46 ± 31.15
Daily intake of phosphorus (mg)	962.27 ± 446.42
Education level, n (%)	
Elementary school and below	8204 (27.26%)
Middle school	3078 (10.23%)
High school	9350 (31.07%)
College and above	9461 (31.44%)
Household income, n (%)	
Low	5994 (19.96%)
Medium–low	7609 (25.34%)
Medium–high	8058 (26.84%)
High	8365 (27.86%)
Economic activity, n (%)	15,216 (50.53%)
Residential areas, n (%)	
Urban	24,559 (81.31%)
Rural	5645 (18.69%)
Frequency of drinking per year, n (%)	
Never	5279 (17.58%)
Have not drunk in the last 1 year	5529 (18.41%)
Less than once a month	7300 (24.31%)
Once a month	3275 (10.91%)
2–4 times a month	5533 (18.43%)
2–3 times a week	2447 (8.15%)
≥ 4 times a week	662 (2.20%)
Smoking status, n (%)	
Non-smoker	26,793 (89.14%)
Smoker	1575 (5.24%)
Ex-smoker	1688 (5.62%)
Subjective body image, n (%)	
Very skinny	1070 (3.56%)
A bit skinny	2916 (9.70%)
Normal	12,325 (40.98%)
A bit fat	10,654 (35.42%)
Very fat	3110 (10.34%)
Weight change in the last 1 year, n (%)	
Maintained	18,872 (62.82%)
Lost	3817 (12.71%)
Gained	7353 (24.48%)
The days of weight training per week, n (%)	
0 day	24,997 (83.05%)
1 day	1037 (3.45%)
2 days	1265 (4.20%)
3 days	1178 (3.91%)
4 days	507 (1.68%)
≥ 5 days	1116 (3.71%)
EQ-5D index	0.93 ± 0.13 (1.00)
Stress awareness, n (%)	
Feel a great deal of stress	1522 (5.07%)
Feel much stress	6845 (22.78%)
Feel some stress	17,026 (56.66%)
Feel almost no stress	4654 (15.49%)
Feeling depression in the last 1 year, n (%)	3192 (10.60%)
Medical checkup in the last 2 years, n (%)	18,958 (62.86%)

Values are mean ± standard deviation (median) or n (%).

LDL, low-density lipoprotein; HDL, high-density lipoprotein; EQ-5D, European Quality of Life-5 Dimensions.

### Prediction model for metabolic syndrome

The performance measures for the six prediction models for metabolic syndrome are summarized in Table 2. Among the six prediction models for metabolic syndrome, the random forest performed the best in terms of the area under the receiver operating characteristic curve (AUC); 90.7% (all participants), 87.7% (diagnosed with CVD), and 82.6% (no CVD diagnosis). The values and ranks of the random forest variable importance are summarized in Table 3. A predictor with the ranking of 26th or higher can be considered to be a major predictor in this study, given that it is a top 30% among 86 predictors here. According to the random forest variable importance in Table 3, the major predictors of metabolic syndrome were body mass index (BMI) (0.1032), use of antihypertensive drugs (0.0552), hypertension (0.0499), CVD (0.0453), age at enrollment (0.0437), white blood cell count (0.0297), low-density lipoprotein (LDL), cholesterol levels (0.0263), menstrual status (0.0247), use of lipid-lowering agents (0.0237), red blood cell count (0.0231), total cholesterol levels (0.0229), subjective body image (0.0221), education level (0.0214), daily fat intake (0.0198), hematocrit levels (0.0197), and breastfeeding duration (0.0191). Breastfeeding duration was a major predictor of metabolic syndrome. Let us take an example in which the random forest variable importance of BMI, CVD, or breastfeeding duration is 0.1032, 0.0453, or 0.0191, respectively. Here, the accuracy of the model will decrease by 10.32%, 4.53%, or 1.91% if the values of BMI, CVD, or breastfeeding duration are randomly permutated (or shuffled). The importance rankings of some major predictors showed dramatic changes in the subgroup analysis, i.e., between the participants with and without CVD. For example, the predictors of medication and diagnosis for hypertension ranked second and third for all participants, respectively, but these predictors went out of the top-30 ranking for both subgroups in Table 3. Likewise, the respective rankings of menstrual status and education were eighth and 13th for all the participants, but their rankings dropped to 23rd or lower for both the subgroups in the same table. Breastfeeding duration ranked 16th as a predictor for all the participants. However, it was ranked slightly higher at 14th for those without CVD and much lower at 26th for those with the condition.

**Table 2:** Model performance: the average was measured for 50 runs.

Model	All Participants		CVD undiagnosed		CVD diagnosed	
	Accuracy	AUC	Accuracy	AUC	Accuracy	AUC
LR	0.7727	0.8176	0.8712	0.8878	0.6922	0.8254
DT	0.7825	0.7347	0.8083	0.6553	0.6794	0.6469
NB	0.7954	0.8405	0.7995	0.7885	0.7682	0.7535
RF	0.8442	0.9065	0.8663	0.8765	0.6817	0.8260
SVM	0.7163	0.7964	0.8392	0.5283	0.6800	0.4989
ANN	0.6684	0.5319	0.7590	0.5135	0.8254	0.4986

CVD: cardiovascular disease; AUC: area under the receiver operating characteristic curve; LR: logistic regression; DT: decision tree; NB: naïve Bayes; RF: random forest; SVM: support vector machine; ANN: artificial neural network.

**Table 3:** The variable importance from the Random Forest in predicting metabolic syndrome.

All participants		Value	Rank	CVD undiagnosed		Value	Rank	CVD diagnosed		Value	Rank
v030	BMI	0.1032	1	v030	BMI	0.1266	1	v030	BMI	0.1177	1
v090	Antihypertensive drug	0.0552	2	v005	Age	0.0453	2	v036	WBC counts (Thous/µL)	0.0459	2
v053	Hypertension	0.0499	3	v036	WBC counts (Thous/µL)	0.0353	3	v075	LDL	0.0379	3
v049	CVD	0.0453	4	v075	LDL	0.0330	4	v031	Total cholesterol (mg/dL)	0.0299	4
v005	Age	0.0437	5	v014	Subjective body image	0.0316	5	v091	Lipid-lowering agent	0.0274	5
v036	WBC counts (Thous/µL)	0.0297	6	v031	Total cholesterol (mg/dL)	0.0303	6	v037	RBC counts (Mil/µL)	0.0260	6
v075	LDL	0.0263	7	v037	RBC counts (Mil/µL)	0.0302	7	v041	Daily intake of fat (g)	0.0256	7
v076	Menstrual status	0.0247	8	v033	Hematocrit (%)	0.0263	8	v014	Subjective body image	0.0252	8
v091	Lipid-lowering agent	0.0237	9	v041	Daily intake of fat (g)	0.0255	9	v046	Daily intake of sodium (mg)	0.0250	9
v037	RBC counts (Mil/µL)	0.0231	10	v032	Hemoglobin (g/dL)	0.0251	10	v005	Age	0.0250	10
v031	Total cholesterol (mg/dL)	0.0229	11	v039	Daily intake of water (g)	0.0242	11	v043	Daily intake of calcium (mg)	0.0249	11
v014	Subjective body image	0.0221	12	v046	Daily intake of sodium (mg)	0.0239	12	v048	Daily intake of vitamin C (mg)	0.0246	12
v007	Education level	0.0214	13	v048	Daily intake of vitamin C (mg)	0.0237	13	v039	Daily intake of water (g)	0.0244	13
v041	Daily intake of fat (g)	0.0198	14	v082	BF durations (month)	0.0235	14	v042	Daily intake of carbohydrate (g)	0.0240	14
v033	Hematocrit (%)	0.0197	15	v035	Serum creatinine (mg/dL)	0.0233	15	v033	Hematocrit (%)	0.0236	15
v082	BF duration (month)	0.0191	16	v043	Daily intake of calcium (mg)	0.0231	16	v038	Daily intake of calories (kcal)	0.0234	16
v092	Drug treatment for glucose control	0.0190	17	v042	Daily intake of carbohydrate (g)	0.0230	17	v035	Serum creatinine (mg/dL)	0.0234	17
v032	Hemoglobin (g/dL)	0.0186	18	v045	Daily intake of iron (mg)	0.0227	18	v045	Daily intake of iron (mg)	0.0233	18
v039	Daily intake of water (g)	0.0186	19	v040	Daily intake of protein (g)	0.0223	19	v040	Daily intake of protein (g)	0.0231	19
v048	Daily intake of vitamin C (mg)	0.0184	20	v038	Daily intake of calories (kcal)	0.0222	20	v047	Daily intake of potassium (mg)	0.0231	20
v046	Daily intake of sodium (mg)	0.0183	21	v047	Daily intake of potassium (mg)	0.0222	21	v044	Daily intake of phosphorus (mg)	0.0228	21
v043	Daily intake of calcium (mg)	0.0181	22	v044	Daily intake of phosphorus (mg)	0.0218	22	v092	Drug treatment for glucose control	0.0225	22
v035	Serum creatinine (mg/dL)	0.0180	23	v007	Education level	0.0196	23	v032	Hemoglobin (g/dL)	0.0218	23
v042	Daily intake of carbohydrate (g)	0.0177	24	v034	Blood urea nitrogen (mg/dL)	0.0188	24	v034	Blood urea nitrogen (mg/dL)	0.0204	24
v045	Daily intake of iron (mg)	0.0173	25	v077	Age at menarche (years)	0.0170	25	v094	Oral hypoglycemic agents	0.0204	25
v047	Daily intake of potassium (mg)	0.0172	26	v083	Gravidity	0.0159	26	v082	BF durations (month)	0.0196	26
v040	Daily intake of protein (g)	0.0172	27	v076	Menstrual status	0.0158	27	v093	Insulin	0.0193	27
v038	Daily intake of calories (kcal)	0.0172	28	v092	Drug treatment for glucose control	0.0153	28	v002	Age at enrollment (years)	0.0158	28
v094	Oral hypoglycemic agents	0.0171	29	v094	Oral hypoglycemic agents	0.0152	29	v077	Age at menarche (years)	0.0153	29
v044	Daily intake of phosphorus (mg)	0.0171	30	v002	Age at enrollment (years)	0.0148	30	v011	EQ-5D	0.0146	30
v093	Insulin	0.0171	31	v093	Insulin	0.0139	31	v083	Gravidity	0.0144	31
v034	Blood urea nitrogen (mg/dL)	0.0154	32	v081	Number of breastfed children	0.0137	32	v090	Antihypertensive drug	0.0125	32
v077	Age at menarche (years)	0.0136	33	v091	Lipid-lowering agent	0.0130	33	v081	Number of breastfed children	0.0123	33
v083	Gravidity	0.0135	34	v052	Frequency of drinking per year	0.0127	34	v052	Frequency of drinking per year	0.0119	34
v081	Number of breastfed children	0.0128	35	v011	EQ-5D	0.0124	35	v007	Education level	0.0100	35
v011	EQ-5D	0.0119	36	v006	Household income	0.0107	36	v006	Household income	0.0093	36
v002	Age at enrollment (years)	0.0116	37	v013	Occupation	0.0098	37	v016	Weight control in the last 1 year	0.0087	37
v052	Frequency of drinking per year	0.0100	38	v016	Weight control in the last 1 year	0.0080	38	v017	Stress awareness	0.0084	38
v006	Household income	0.0092	39	v017	Stress awareness	0.0078	39	v013	Occupation	0.0080	39
v013	Occupation	0.0072	40	v015	Weight change in the last 1 year	0.0061	40	v015	Weight change in the last 1 year	0.0056	40
v016	Weight control in the last 1 year	0.0067	41	v018	The days of weight training per week	0.0054	41	v018	The days of weight training per week	0.0048	41
v017	Stress awareness	0.0061	42	v057	Osteoarthritis	0.0040	42	v009	Participation in health examination	0.0040	42
v057	Osteoarthritis	0.0049	43	v010	Cancer screening for the last 2 years	0.0039	43	v021	Diagnosis of HTN in mother	0.0039	43
v015	Weight change in the last 1 year	0.0044	44	v012	Economic activity	0.0038	44	v057	Osteoarthritis	0.0038	44
v018	The days of weight training per week	0.0041	45	v009	Participation in health examination	0.0037	45	v010	Cancer screening for the last 2 years	0.0037	45
v010	Cancer screening for the last 2 years	0.0030	46	v051	Smoking	0.0035	46	v053	Hypertension	0.0037	46
v009	Participation in health examination	0.0029	47	v021	Diagnosis of HTN in mother	0.0035	47	v012	Economic activity	0.0036	47
v012	Economic activity	0.0028	48	v003	Residental area (urban/rural)	0.0034	48	v019	Use of oral contraceptive	0.0036	48
v003	Residental area (urban/rural)	0.0027	49	v029	Diagnosis of DM in mother	0.0032	49	v003	Residental area (urban/rural)	0.0036	49
v019	Use of oral contraceptive	0.0026	50	v019	Use of oral contraceptive	0.0032	50	v076	Menstrual status	0.0035	50
v021	Diagnosis of HTN in mother	0.0026	51	v020	Diagnosis of HTN in father	0.0027	51	v062	Depression	0.0032	51
v051	Smoking	0.0023	52	v074	Melancholy in the last 1 year	0.0027	52	v020	Diagnosis of HTN in father	0.0030	52
v020	Diagnosis of HTN in father	0.0022	53	v062	Depression	0.0026	53	v074	Melancholy in the last 1 year	0.0029	53
v074	Melancholy in the last 1 year	0.0021	54	v028	Diagnosis of DM in father	0.0024	54	v027	Diagnosis of stroke in mother	0.0026	54
v080	History of breastfeeding	0.0021	55	v080	History of breastfeeding	0.0023	55	v026	Diagnosis of stroke in father	0.0025	55
v062	Depression	0.0020	56	v027	Diagnosis of stroke in mother	0.0021	56	v051	Smoking	0.0025	56
v029	Diagnosis of DM in mother	0.0020	57	v026	Diagnosis of stroke in father	0.0020	57	v056	Stroke	0.0024	57
v027	Diagnosis of stroke in mother	0.0017	58	v061	Thyroid disease	0.0020	58	v055	angina	0.0023	58
v079	Childbirth experience	0.0017	59	v079	Childbirth experience	0.0018	59	v061	Thyroid disease	0.0022	59
v026	Diagnosis of stroke in father	0.0016	60	v008	Marriage	0.0017	60	v058	Rheumatic arthritis	0.0020	60
v028	Diagnosis of DM in father	0.0016	61	v060	Asthma	0.0017	61	v029	Diagnosis of DM in mother	0.0020	61
v061	Thyroid disease	0.0015	62	v078	Pregnancy experience	0.0015	62	v080	History of breastfeeding	0.0019	62
v078	Pregnancy experience	0.0013	63	v059	Tuberculosis	0.0014	63	v059	Tuberculosis	0.0019	63
v008	Marriage	0.0012	64	v064	Atopic dermatitis	0.0012	64	v060	Asthma	0.0015	64
v060	Asthma	0.0012	65	v024	Diagnosis of IHD in father	0.0012	65	v028	Diagnosis of DM in father	0.0013	65
v059	Tuberculosis	0.0011	66	v058	Rheumatic arthritis	0.0012	66	v025	Diagnosis of IHD in mother	0.0011	66
v058	Rheumatic arthritis	0.0011	67	v023	Diagnosis of hyperlipidemia in mother	0.0012	67	v023	Diagnosis of hyperlipidemia in mother	0.0010	67
v056	Stroke	0.0011	68	v025	Diagnosis of IHD in mother	0.0011	68	v079	Childbirth experience	0.0009	68
v023	Diagnosis of hyperlipidemia in mother	0.0009	69	v056	Stroke	0.0010	69	v064	Atopic dermatitis	0.0008	69
v024	Diagnosis of IHD in father	0.0009	70	v068	Breast cancer	0.0006	70	v054	Myocardial infarction	0.0008	70
v055	Angina	0.0009	71	v022	Diagnosis of hyperlipidemia in father	0.0006	71	v065	Gastric cancer	0.0007	71
v025	Diagnosis of IHD in mother	0.0008	72	v069	Cervical cancer	0.0005	72	v024	Diagnosis of IHD in father	0.0006	72
v064	Atopic dermatitis	0.0007	73	v071	Hepatitis B	0.0004	73	v078	Pregnancy experience	0.0006	73
v068	Breast cancer	0.0005	74	v067	Colon cancer	0.0004	74	v069	Cervical cancer	0.0006	74
v069	Cervical cancer	0.0004	75	v072	Hepatitis C	0.0002	75	v068	Breast cancer	0.0006	75
v022	Diagnosis of hyperlipidemia in father	0.0004	76	v073	Liver cirrhosis	0.0001	76	v073	Liver cirrhosis	0.0005	76
v071	Hepatitis B	0.0004	77	v065	Gastric cancer	0.0001	77	v008	Marriage	0.0005	77
v067	Colon cancer	0.0003	78	v063	Chronic kidney disease	0.0001	78	v071	Hepatitis B	0.0005	78
v065	Gastric cancer	0.0003	79	v070	Lung cancer	0.0001	79	v063	Chronic kidney disease	0.0005	79
v054	Myocardial infarction	0.0003	80	v066	Liver cancer	0.0000	80	v067	Colon cancer	0.0003	80
v073	Liver cirrhosis	0.0002	81	v004	Sex	0.0000	81	v022	Diagnosis of hyperlipidemia in father	0.0003	81
v063	Chronic kidney disease	0.0002	82	v049	CVD	0.0000	81	v072	Hepatitis C	0.0002	82
v072	Hepatitis C	0.0002	83	v053	Hypertension	0.0000	81	v070	Lung cancer	0.0001	83
v070	Lung cancer	0.0001	84	v054	Myocardial infarction	0.0000	81	v066	Liver cancer	0.0000	84
v066	Liver cancer	0.0000	85	v055	Angina	0.0000	81	v004	Sex	0.0000	85
v004	Sex	0.0000	86	v090	Antihypertensive drug	0.0000	81	v049	CVD	0.0000	85

TG, triglyceride; LDL, low-density lipoprotein; HDL, high-density lipoprotein; BMI, body mass index; IHD, ischemic heart disease; DM, diabetes mellitus; HTN, hypertension; WBC, white blood cell; CVD, cardiovascular disease; RBC, red blood cell; EQ-5D, European Quality of Life-5 Dimension.

The logistic analysis results for each important variable, including obstetric characteristics, are presented in Supplementary Material 2. The breastfeeding duration was associated with a decreased risk of metabolic syndrome (adjusted odds ratio [aOR] 0.998; confidence interval [CI] [0.996–1.000]). The odds of metabolic syndrome will decrease by 0.2% if breastfeeding duration increases by 1 month. In other words, the odds of metabolic syndrome will decrease by 2.4% (or 4.8%) if breastfeeding duration increases by 1 year, i.e., 12 months (or 2 years, i.e., 24 months). The effect of breastfeeding duration on metabolic syndrome looks small on 1 month but it is big on 1 year or two. The odds ratio is not statistical significant at 5% level but it is still useful information in machine learning, given that variable importance is primary and statistical significance is supplementary in machine learning. Logistic regression requires adopting the unrealistic assumption of *ceteris paribus*, i.e., “all the other variables remain constant”. In this context, the results of the logistic regression would serve as supplementary information to the random forest variable importance.

## Discussion

In summary, among the obstetric characteristics, one of the most significant factors associated with metabolic syndrome was the duration of breastfeeding. Among the six prediction models for metabolic syndrome, the random forest had the best performance in terms of the AUC, i.e., 90.7% (all participants). In the subgroup analysis, among the women without CVD, the importance of breastfeeding duration as a predictor of metabolic syndrome was ranked 14th (0.0235), which is as important as the daily intake of sodium (12th, 0.0239).

This study presents the most comprehensive analysis of the determinants of metabolic syndrome in women using a large-scale Asian population-based cross-sectional study of 30,204 participants. While there is one paper that has addressed the association between breastfeeding and metabolic syndrome in postmenopausal women using KHANES data, our study differs in that it targeted all adult women, included more recent data (2010 to 2018), and distinguished itself by constructing a predictive model for metabolic syndrome using machine learning^9^. This study investigated whether there were differences in metabolic syndrome-related factors between the women with and without CVD. In a recent meta-analysis, the authors assumed that breastfeeding may have a preventive effect on metabolic syndrome and that it was related to breastfeeding duration^8^. However, the pooled effect of breastfeeding on metabolic syndrome was not conclusive because of the study population heterogeneity, the criteria for breastfeeding, and confounding factors for metabolic syndrome^8^. In this large-scale population-based study, we evaluated the precise impact of breastfeeding on metabolic syndrome and compared its clinical importance to the other known risk factors known to predispose women to metabolic syndrome.

During pregnancy, the mother undergoes metabolic changes that increase insulin resistance and serum lipid levels (particularly triglyceride [TG])^21,22^. Breastfeeding reportedly restores the overall maternal postpartum metabolic changes faster back to the prenatal baselines^23^. It also has a long-term positive effect on maternal glucose levels, lipid metabolism, and adiposity^23–25^. The relationship between gravidity, parity, and metabolic syndrome is still debated, necessitating further research.

In this study, we investigated the importance of specific variables in the development of metabolic syndrome in women with and without CVD. The relative importance of different variables between the participants with and without CVD can have important clinical implications. First, in women without CVD, age (second vs. tenth), breastfeeding duration (14th vs. 26th), and gravidity (26th vs. 31st) were ranked higher as compared to women with CVD. These variables appeared to have a higher association with metabolic syndrome in the women without CVD and were less important in women with CVD. Second, in women with CVD, the importance of lipid-lowering agents or diabetes drugs was relatively higher. A previous meta-analysis reported that among the five factors of metabolic syndrome, the prognosis of CVD was especially poor in patients with dyslipidemia or impaired glucose tolerance^26^. In this study, it can also be hypothesized that dyslipidemia or impaired glucose tolerance has a stronger mediating effect on metabolic syndrome in women with CVD. Third, in the three models of this study (Table 3), the nutrient intake (especially fat intake) was highly correlated with metabolic syndrome, and the importance of nutrient intake was higher in women with CVD than in women without CVD. Previous studies have reported the significance of healthy diets for metabolic syndrome, which was further emphasized in this study^27^. Moreover, the importance of diet in metabolic syndrome was reported to be greater in women with CVD than in women without CVD. Additionally, white blood cell count ranked sixth or higher as a predictor of metabolic syndrome in women. Levels of C-reactive protein, plasma, and low-grade inflammation have been reported to be positively associated with metabolic syndrome^28,29^. It is reasonable to speculate that the white blood cell count also has a positive relationship with metabolic syndrome.

This study has limitations. First, a cross-sectional design was used. However, using data with a longitudinal design is expected to improve the validity of this study. Second, the duration of breastfeeding in this study is reliant on information that has been self-reported several years after the actual breastfeeding took place, which may introduce limitations to the accuracy of the data. Furthermore, although the medical history was presumed based on a physician's diagnosis, it may be subject to limitations in accuracy as it relied on self-report surveys by the participants. Similarly, an investigation into dietary intake involved a nutritionist conducting direct interviews during visits. However, there may be limitations to the objectivity of respondents' responses. Third, expanding this study to other diseases and predictors such as health utility usage might significantly contribute to this line of research. Fourth, we excluded the diagnostic criteria for the metabolic syndrome from the independent variables. However, to examine the influence of CVD and the use of cardiovascular medications on the metabolic syndrome, we included the presence of hypertension diagnosed by a physician and the use of cardiovascular medications as independent variables. Fifth, this study used random forest variable importance as primary results and logistic regression odds ratios as supplementary findings. That is, the former result was considered to be the strength of the association between metabolic syndrome and its major predictor, while the latter finding was considered to be the direction of the association. There would be other ways to examine the direction of the association, and this would make a great contribution for research in this direction. Finally, this study did not consider the possible mediating effects among the variables.

In the prediction model with a random forest of AUC 90.7%, the top predictors of metabolic syndrome included body mass index (0.1032), medication for hypertension (0.0552), hypertension (0.0499), cardiovascular disease (0.0453), age (0.0437) and breastfeeding duration (0.0191). Breastfeeding duration was one of the most important predictors of metabolic syndrome among the various obstetric characteristics.

## Methods

### Study population

This study was based on the fifth (2010–2012), sixth (2013–2015), seventh (2016–2018), and eighth (2019) Korean National Health and Nutrition Examination Survey (KNHANES) surveys. The KNHANES is a nationwide representative survey that obtains samples annually using a stratified multistage cluster sampling design. The KHANSE is conducted by a dedicated research team, visiting four regions each week (for a total of 192 regions annually). The survey is conducted over a period of 3 days in each region, with mobile examination vehicles visiting the area to perform health screenings, health surveys, and nutritional assessments. Health surveys and medical examinations are conducted in mobile examination vehicles, while nutritional assessments are performed by a specialized team of nutritionists who visit households directly. This data is used to assess the health status, prevalence of chronic diseases, and nutritional intake status of the population in South Korea. In the KNHANES 2010–2019, men and participants under the age of 20 years were excluded from the current analyses. The cases with missing data on the chronic occurrence or diagnosis of hypertension, myocardial infarction, angina, all the factors associated with the diagnosis of metabolic syndrome, and an outlier (the woman over 80 years old before menarche) were excluded.

The data were publicly available and de-identified. The requirement for ethical approval was waived by the institutional review board of Korea University Anam Hospital. All methods were conducted in accordance with relevant institutional/ethical committee guidelines and regulations. The requirement for informed consent was waived because all participant information was deidentified and encrypted to protect privacy.

### Variables

The variables included in this study are summarized in Supplementary Materials 1. The sociodemographic characteristics, including the age at enrollment, sex, body mass index (BMI), household income (represented as quartiles), marital status, the level of education (elementary school and below, middle school, high school, and college and above), areas of residence, economic activities, and occupations, were assessed using questionnaires.

Information regarding the general obstetric characteristics, including gravidity, parity, breastfeeding (history of breasting, the number of children breastfed, and lifetime total breastfeeding duration), history of abortions, the age at menarche, and the menstrual status (menstruation, pregnancy, breastfeeding, menopause, and others), were also obtained from the questionnaires. The presence of the following diseases was defined based on an interview: (1) hypertension, (2) myocardial infarction, (3) angina, (4) stroke, (5) osteoarthritis, (6) rheumatoid arthritis, (7) pulmonary tuberculosis, (8) asthma, (9) thyroid-related disease, (10) major depressive disorder, (11) kidney failure, (12) hepatitis B, (13) hepatitis C, (14) liver cirrhosis, (14) cancers (gastric cancer, hepatic cancer, colorectal cancer, breast cancer, cervical cancer, and lung cancer), and (15) atopic dermatitis. Data on family histories of hypertension, hyperlipidemia, ischemic heart disease, stroke, and diabetes mellitus were also obtained from the questionnaires. Additionally, the questionnaires also provided the data on the use of (1) antihypertensive drugs, (2) lipid-lowering agents, (3) oral hypoglycemic agents, and (4) insulin.

The blood pressures, waist circumferences and body mass index (BMI) of the participants were measured. Levels of total cholesterol, TG, LDL, high-density lipoprotein (HDL), hemoglobin, hematocrit, blood urea nitrogen, blood creatinine, white blood cell, and red blood cell were also measured at the time of survey.

The participants answered questions about their insights and habits associated with their health. They were asked about their subjective body image, their goals associated with controlling their body weights, history of medical checkups for the past 2 years, history of smoking, frequency of alcohol consumption (per year), and weekly weight training routines. Data on mental health, including stress awareness and feelings of depression within a year, were also collected. The quality of life, based on health indicators, was assessed using the European Quality of Life-5 Dimensions (EQ-5D) scale^30^. The daily intake of energy (kcal), carbohydrates (g), protein (g), fat (g), sodium (mg), water (g), calcium (mg), phosphorus (mg), iron (mg), potassium (mg), and vitamin C (mg) was ascertained from the nutrition survey.

A diagnosis for CVD required the presence of at least one of the following: (1) hypertension, (2) myocardial infarction, or (3) angina. Based on the modified National Cholesterol Rationale Education Program Adult Treatment Program III criteria and the appropriate cutoff for central obesity in Korean adult women (suggested by the Korean Endocrine Society), metabolic syndrome was defined as having three or more of the following^1,31^: (1) central obesity (waist circumference ≥ 85 cm); (2) elevated TGs (serum TG concentration ≥ 150 mg/dL); (3) low HDL cholesterol (serum HDL cholesterol concentration < 50 mg/dL); (4) elevated blood pressure (systolic blood pressure ≥ 130 mmHg or diastolic blood pressure ≥ 85 mmHg) or the prescription of antihypertensive drugs; (5) elevated fasting glucose (fasting serum glucose ≥ 100 mg/dL) or the prescription of diabetes drugs. And we excluded the variables corresponding to the diagnostic criteria of metabolic syndrome among the independent variables, including waist circumference, TG, HDL cholesterol, blood pressure measurements, and fasting glucose.

### Statistical analysis

An artificial neural network, decision tree, logistic regression, naïve Bayes, random forest, and support vector machine were used to predict metabolic syndrome. Data on 30,204 observations with full information were divided into training and validation sets in a 70:30 ratio (21,143:9061). The AUC curve and accuracy (the ratio of correct predictions among the 9061 observations in the validation set) were employed as the standard for model validation. The random forest variable importance, the contribution of a certain variable to the random forest performance (accuracy), was used to examine the major predictors of metabolic syndrome. Let us assume that the importance of the random forest variable of CVD is 0.0453. Here, the accuracy of the model drops by 4.53% if the values of a predictor of CVD are randomly permutated (or shuffled). The random split and analysis were repeated 50 times and averaged for external validation^32–34^. R-Studio 1.3.959 (R-Studio Inc.: Boston, United States) and Python 3.52 (CreateSpace: Scotts Valley, United States) were employed for the analysis between February 1, 2022–March 31, 2022.

### Supplementary Information

Supplementary Information.

## Data Availability

The data utilized in this study is available from the Korean National Health and Nutrition Examination Survey (KNHANES) (https://knhanes.kdca.go.kr/knhanes). The datasets used and/or analyzed during the current study available from the corresponding author on reasonable request.
