# Author Correction: Machine learning analysis for the association between breast feeding and metabolic syndrome in women

**DOI:** 10.1038/s41598-024-57571-4

**Published:** 2024-03-26

**Authors:** Jue Seong Lee, Eun-Saem Choi, Hwasun Lee, Serhim Son, Kwang-Sig Lee, Ki Hoon Ahn

**Affiliations:** 1 Department of Pediatrics, Korea University College of Medicine, Korea University Anam Hospital, Seoul, South Korea; 2 Department of Obstetrics and Gynecology, Korea University College of Medicine, Korea University Anam Hospital, 73 Goryeodae-ro, Seongbuk-gu, Seoul, 02841 South Korea; 3 Department of Biostatistics, Korea University College of Medicine, Seoul, South Korea; 4 AI Center, Korea University College of Medicine, Korea University Anam Hospital, 73 Goryeodae-ro, Seongbuk-gu, Seoul, 02841 South Korea

Correction to: *Scientific Reports* 10.1038/s41598-024-53137-6, published online 20 February 2024

The Funding section in the original version of this Article was omitted. The Funding section now reads:

“This work was supported by (1) the Korea University Medical Center grant (No. K1925051; Author Ki Hoon Ahn; https://www.kumc.or.kr/en/index.do), (2) the Korea Health Industry Development Institute grant (Korea Health Technology R&D Project) funded by the Ministry of Health & Welfare of South Korea (No. HI22C1463; Author Ki Hoon Ahn; https://www.khidi.or.kr/eps), and technically supported by 4P Lab, Co., Ltd for data analysis (Authors Ki Hoon Ahn & Kwang-Sig Lee), and (3) the Korea Health Industry Development Institute grant (Korea Health Technology R&D Project) funded by the Ministry of Health & Welfare of South Korea (No. HI22C1302; Author Kwang-Sig Lee; https://www.khidi.or.kr/eps). There was no additional external funding received for this study. The funders had no role in study design, data collection and analysis, decision to publish, or preparation of the manuscript.”

The original Article has been corrected.

