# Supplementary material for: Machine learning analysis for the association between breast feeding and metabolic syndrome in women
Source: Sci Rep. 2024 Feb 20;14:4138. doi: 10.1038/s41598-024-53137-6 (PMC10876622; doi:10.1038/s41598-024-53137-6)
Supplement: Supplementary file 1 — Supplementary Information. [file 41598_2024_53137_MOESM1_ESM.docx]

**Supplementary Materials**

**Supplementary Materials 1.** Variable description

**Supplementary Materials 2.** Multivariate logistic regression analysis of the association between metabolic syndrome and variables

**Supplementary Materials 1.** Variable description

| **Variable** | **Code** | **Missing** |
| --- | --- | --- |
| ID | v001 | 0.00 |
| Metabolic syndrome | v050 | 0.00 |
| Age at enrollment (years) | v002 | 0.00 |
| Residental area (urban/rural) | v003 | 0.00 |
| Sex | v004 | 0.00 |
| Age | v005 | 0.00 |
| Household income | v006 | 0.01 |
| Education level | v007 | 0.00 |
| Marriage | v008 | 0.00 |
| Participation in health examination | v009 | 0.00 |
| Cancer screening for last 2 years | v010 | 0.00 |
| EQ5D | v011 | 0.00 |
| Economic activity | v012 | 0.00 |
| Occupation | v013 | 0.00 |
| Subjective body image | v014 | 0.00 |
| Weight change in the last 1 year | v015 | 0.01 |
| Weight control in the last 1 year | v016 | 0.00 |
| Stress awareness | v017 | 0.01 |
| The days of weight training per week | v018 | 0.00 |
| Use of oral contraceptive | v019 | 0.00 |
| Diagnosis of HTN in father | v020 | 0.09 |
| Diagnosis of HTN in mother | v021 | 0.06 |
| Diagnosis of hyperlipidemia in father | v022 | 0.10 |
| Diagnosis of hyperlipidemia in mother | v023 | 0.08 |
| Diagnosis of IHD in father | v024 | 0.09 |
| Diagnosis of IHD in mother | v025 | 0.07 |
| Diagnosis of stroke in father | v026 | 0.08 |
| Diagnosis of stroke in mother | v027 | 0.06 |
| Diagnosis of DM in father | v028 | 0.08 |
| Diagnosis of DM in mother | v029 | 0.06 |
| BMI | v030 | 0.00 |
| Total cholesterol (mg/dL) | v031 | 0.00 |
| Hemoglobin (g/dL) | v032 | 0.00 |
| Hematocrit (%) | v033 | 0.00 |
| Blood urea nitrogen (mg/dL | v034 | 0.00 |
| Serum creatinine (mg/dL) | v035 | 0.00 |
| White blood cell counts (Thous/µL) | v036 | 0.00 |
| Red blood cell counts (Mil/µL) | v037 | 0.00 |
| Daily intake of calorie (kcal) | v038 | 0.09 |
| Daily intake of water (g) | v039 | 0.09 |
| Daily intake of protein (g) | v040 | 0.09 |
| Daily intake of fat (g) | v041 | 0.09 |
| Daily intake of carbohydrate (g) | v042 | 0.09 |
| Daily intake of calcium (mg) | v043 | 0.09 |
| Daily intake of phosphorus (mg) | v044 | 0.09 |
| Daily intake of iron (mg) | v045 | 0.09 |
| Daily intake of sodium (mg) | v046 | 0.09 |
| Daily intake of potassium (mg) | v047 | 0.09 |
| Daily intake of vitamin C (mg) | v048 | 0.09 |
| Cardiovascular disease | v049 | 0.00 |
| Smoking | v051 | 0.00 |
| Frequency of drinking per year | v052 | 0.01 |
| Hypertension | v053 | 0.00 |
| Myocardial infarction | v054 | 0.00 |
| Angina | v055 | 0.00 |
| Stroke | v056 | 0.00 |
| Osteoarthritis | v057 | 0.00 |
| Rheumatic arthritis | v058 | 0.00 |
| Tuberculosis | v059 | 0.00 |
| Asthma | v060 | 0.00 |
| Thyroid disease | v061 | 0.00 |
| Depression | v062 | 0.00 |
| Chronic kidney disease | v063 | 0.00 |
| Atopic dermatitis | v064 | 0.00 |
| Gastric cancer | v065 | 0.00 |
| Liver cancer | v066 | 0.00 |
| Colon cancer | v067 | 0.00 |
| Breast cancer | v068 | 0.00 |
| Cervical cancer | v069 | 0.00 |
| Lung cancer | v070 | 0.00 |
| Hepatitis B | v071 | 0.00 |
| Hepatitis C | v072 | 0.00 |
| Liver cirrhosis | v073 | 0.00 |
| Melancholy in the last 1 year | v074 | 0.00 |
| Serum LDL cholesterol (mg/dL) | v075 | 0.00 |
| Menstrual status | v076 | 0.01 |
| Age at menarche (years) | v077 | 0.01 |
| Pregnancy experience | v078 | 0.01 |
| Childbirth experience | v079 | 0.01 |
| Breastfeeding experience | v080 | 0.01 |
| Number of breastfed children | v081 | 0.01 |
| Breastfeeding duration (months) | v082 | 0.01 |
| Gravidity | v083 | 0.01 |
| Waist circumference (cm) | v084 | 0.00 |
| Serum TG (mg/dL) | v085 | 0.00 |
| Serum HDL cholesterol (mg/dL) | v086 | 0.00 |
| Systolic blood pressure (mmHg) | v087 | 0.00 |
| Diastolic blood pressure (mmHg) | v088 | 0.00 |
| Serum fasting glucose (mg/dL) | v089 | 0.00 |
| Antihypertensive drug | v090 | 0.00 |
| Lipid-lowering agent | v091 | 0.00 |
| Drug treatment for glucose control | v092 | 0.00 |
| Insulin | v093 | 0.00 |
| Oral hypoglycemic agents | v094 | 0.00 |

EQ-5D, European Quality of Life-5 Dimensions; HTN, hypertension; IHD, ischemic heart disease; DM, diabetes mellitus; BMI, body mass index; LDL, low-density lipoprotein; TG, triglyceride; HDL, high-density lipoprotein.

| **Variable** |  |
| --- | --- |
| ID |  |
| Year |  |
| Residental area | 1. Urban |
|  | 2. Rural |
| Sex | 1. Male |
|  | 2. Female |
| Age |  |
| Household income | 1. Lowest 25 percentile |
|  | 2. 25.1–50 percentile |
|  | 3. 50.1–75 percentile |
|  | 4. Highest 25 percentile |
| Education level | 1. Less than elementary school graduation |
|  | 2. Middle School graduation |
|  | 3. High School graduation |
|  | 4. College graduate or higher |
| Marriage | 1. Married |
|  | 2. Single |
| Participation in health examination | 1. yes |
|  | 2. no |
| Cancer screening for last 2 years | 1. yes |
|  | 2. no |
| EQ5D | □.□□□ |
| Economic activity | 1. yes |
|  | 2. no |
| Occupation | 1. Manager |
|  | 2. Professionals and related practitioners |
|  | 3. Office worker |
|  | 4. Service worker |
|  | 5. Sales person |
|  | 6. Skilled workers in agriculture, forestry and fishing |
|  | 7. Technicians and related technical workers |
|  | 8. Persons engaged in device, machine operation, and assembly |
|  | 9. Simple labor worker |
|  | 10. Soldier |
|  | 88. Unemployed, economically inactive population |
| Subjective body image | 1. Very skinny |
|  | 2. Slightly skinny |
|  | 3. Average |
|  | 4. Slightly overweight |
|  | 5. Very obese |
| Weight change in the last 1 year | 1. No change |
|  | 2. Weight loss |
|  | 3. Weight gain |
| Weight control in the last 1 year | 1. Weight loss efforts |
|  | 2. Weight maintenance effort |
|  | 3. Weight gain effort |
|  | 4. Never tried to weight control |
| Stress awareness | 1. Feel very stressed |
|  | 2. Feel a lot of stress |
|  | 3. Feel a little stressed |
|  | 4. Hardly feel any stress |
| The days of weight training per week | 1. Not at all |
|  | 2. 1 day |
|  | 3. 2 day |
|  | 4. 3 day |
|  | 5. 4 day |
|  | 6. More than 5 days |
| Use of oral contraceptive | 1. yes |
|  | 2. no |
| Diagnosis of HTN in father | 0. no |
|  | 1. yes |
| Diagnosis of HTN in mother | 0. no |
|  | 1. yes |
| Diagnosis of hyperlipidemia in father | 0. no |
|  | 1. yes |
| Diagnosis of hyperlipidemia in mother | 0. no |
|  | 1. yes |
| Diagnosis of IHD in father | 0. no |
|  | 1. yes |
| Diagnosis of IHD in mother | 0. no |
|  | 1. yes |
| Diagnosis of stroke in father | 0. no |
|  | 1. yes |
| Diagnosis of stroke in mother | 0. no |
|  | 1. yes |
| Diagnosis of DM in father | 0. no |
|  | 1. yes |
| Diagnosis of DM in mother | 0. no |
|  | 1. yes |
| BMI | □□□.□ kg/m2 |
| Total cholesterol (mg/dL) | □□□.□ mg/dL |
| Hemoglobin (g/dL) | □□.□ g/dL |
| Hematocrit (%) | □□.□ % |
| Blood urea nitrogen (mg/dL | □□ mg/dL |
| Serum creatinine (mg/dL) | □□.□□ mg/dL |
| White blood cell counts (Thous/µL) | □□.□□ Thous/µL |
| Red blood cell counts (Mil/µL) | □.□□ Mil/µL |
| Daily intake of calorie (kcal) |  |
| Daily intake of water (g) |  |
| Daily intake of protein (g) |  |
| Daily intake of fat (g) |  |
| Daily intake of carbohydrate (g) |  |
| Daily intake of calcium (mg) |  |
| Daily intake of phosphorus (mg) |  |
| Daily intake of iron (mg) |  |
| Daily intake of sodium (mg) |  |
| Daily intake of potassium (mg) |  |
| Daily intake of vitamin C (mg) |  |
| Cardiovascular disease | 0. no |
|  | 1. yes |
| Metabolic syndrome | 0. no |
|  | 1. yes |
| Smoking | 0. Non-smoker |
|  | 1. Smoker |
|  | 2. Ex-smoker |
| Frequency of drinking per year | 0. Never drank |
|  | 1. Haven't drank at all in the last year |
|  | 2. Less than once a month |
|  | 3. About once a month |
|  | 4. 2-4 times a month |
|  | 5. 2-3 times a week |
|  | 6. 4 or more times a week |
| Hypertension | 0. no |
|  | 1. yes |
| Myocardial infarction | 0. no |
|  | 1. yes |
| Angina | 0. no |
|  | 1. yes |
| Stroke | 0. no |
|  | 1. yes |
| Osteoarthritis | 0. no |
|  | 1. yes |
| Rheumatic arthritis | 0. no |
|  | 1. yes |
| Tuberculosis | 0. no |
|  | 1. yes |
| Asthma | 0. no |
|  | 1. yes |
| Thyroid disease | 0. no |
|  | 1. yes |
| Depression | 0. no |
|  | 1. yes |
| Chronic kidney disease | 0. no |
|  | 1. yes |
| Atopic dermatitis | 0. no |
|  | 1. yes |
| Gastric cancer | 0. no |
|  | 1. yes |
| Liver cancer | 0. no |
|  | 1. yes |
| Colon cancer | 0. no |
|  | 1. yes |
| Breast cancer | 0. no |
|  | 1. yes |
| Cervical cancer | 0. no |
|  | 1. yes |
| Lung cancer | 0. no |
|  | 1. yes |
| Hepatitis B | 0. no |
|  | 1. yes |
| Hepatitis C | 0. no |
|  | 1. yes |
| Liver cirrhosis | 0. no |
|  | 1. yes |
| Melancholy in the last 1 year | 0. no |
|  | 1. yes |
| Serum LDL cholesterol (mg/dL) |  |
| Menstrual status | 1. Before menarche |
|  | 2. During menstruation |
|  | 3. Pregnant |
|  | 4. Lactating |
|  | 5. Menopause |
|  | 7. etc. |
| Age at menarche (years) | □□□ years |
| Pregnancy experience | 1. yes |
|  | 2. no |
| Childbirth experience | 1. yes |
|  | 2. no |
|  | 8. No pregnancy experience |
| Breastfeeding experience | 1. yes |
|  | 2. no |
|  | 8. No birth experience |
| Number of breastfed children | □□ |
|  | 0. No breastfeeding experience |
| Breastfeeding duration (months) | □□month |
|  | 0. No breastfeeding experience |
| Gravidity | □□ |
|  | 0. No pregnancy experience |
| Waist circumference (cm) | □□□.□ cm |
| Serum TG (mg/dL) | □□□□.□ mg/dL |
| Serum HDL cholesterol (mg/dL) | □□.□□ mg/dL |
| Systolic blood pressure (mmHg) | □□□ mmHg |
| Diastolic blood pressure (mmHg) | □□□ mmHg |
| Serum fasting glucose (mg/dL) | □□□.□ mg/dL |
| Antihypertensive drug | 1. Taken daily |
|  | 2. Taking more than 20 days per month |
|  | 3.Taken more than 15 days per month |
|  | 4. Taken less than 15 days per month |
|  | 5. Not taking |
|  | 8. Not applicable |
| Lipid-lowering agent | 1. Taken daily |
|  | 2. Taking more than 20 days per month |
|  | 3. Taken more than 15 days per month |
|  | 4. Taken less than 15 days per month |
|  | 5. Not taking |
|  | 8. Not applicable |
| Drug treatment for glucose control | 1. yes |
|  | 2. no |
|  | 8. not applicable |
| Insulin | 0. no |
|  | 1. yes |
|  | 8. not applicable |
| Oral hypoglycemic agents | 0. no |
|  | 1. yes |
|  | 8. not applicable |

EQ-5D, European Quality of Life-5 Dimensions; HTN, hypertension; IHD, ischemic heart disease; DM, diabetes mellitus; BMI, body mass index; LDL, low-density lipoprotein; TG, triglyceride; HDL, high-density lipoprotein.

**Supplementary Materials 2.** Logistic analysis results on metabolic syndrome with important variables including obstetric characteristics

| Variables | Odds ratio | 95% CI | P value |
| --- | --- | --- | --- |
| Age | 1.060 | 1.053 – 1.068 | <0.001 |
| BMI | 1.376 | 1.350 – 1.401 | < 0.001 |
| Hypertension | 4.212 | 2.574 – 6.893 | <0.001 |
| Myocardial infarction | 2.256 | 1.260 – 4.042 | 0.006 |
| CVD | 0.867 | 0.526 – 1.430 | 0.577 |
| LDL | 0.972 | 0.969 – 0.975 | <0.001 |
| Total cholesterol (mg/dL) | 1.020 | 1.018 – 1.023 | <0.001 |
| Age at menarche (years) | 1.022 | 0.999 – 1.045 | 0.059 |
| Parous women | 0.723 | 0.328 – 1.596 | 0.422 |
| Gravidity | 0.995 | 0.971 – 1.019 | 0.680 |
| Use of oral contraceptive | 1.051 | 0.948 – 1.165 | 0.341 |
| Breastfeeding experience | 0.889 | 0.643 – 1.229 | 0.476 |
| Number of breastfed children | 1.022 | 0.964 – 1.083 | 0.465 |
| Breastfeeding duration (month) | 0.998 | 0.996 – 1.000 | 0.058 |

CI, confidence interval; BMI, body mass index; CVD, cardiovascular disease; LDL, low-density lipoprotein.
